# An accidental emamectin benzoate poisoning in child: A case report

**DOI:** 10.1002/ccr3.6133

**Published:** 2022-07-25

**Authors:** Gopal Kumar Yadav, Dipesh Kumar Rohita, Krishna Chandra Mandal, Binod Paudel, Ashok Raj Devkot

**Affiliations:** ^1^ Department of Internal Medicine Kalaiya District Hospital Bara Nepal; ^2^ Department of Internal Medicine BP Koirala Institute of Health Sciences Dharan Nepal; ^3^ Department of Emergency Medicine Gaur District Hospital Rautahat Nepal; ^4^ Department of Emergency Medicine Grahun Primary Hospital Syangja Nepal; ^5^ School of Medicine Brown University Providence Rhode Island USA

**Keywords:** 4′‐deoxy‐4′‐epi‐methyl‐amino benzoate salt, child, emamectin benzoate, poisoning, treatment

## Abstract

We report a case of accidental Emamectin Benzoate poisoning in a six‐year‐old child resulting in nausea, vomiting, abdominal pain, and confusion. We did vigorous gastric lavage with saline, activated charcoal, and coconut oil. The other supportive treatment improved the outcome of the patient with complete resolution of symptoms.

## 1 INTRODUCTION

Emamectin Benzoate (EB, also known as MK‐0244) is the 4′‐deoxy‐4′‐epi‐methyl‐amino benzoate salt of avermectin B1 (avermectin family of 16‐membered macrocyclic lactones), which is similar structurally to natural fermentation products of *Streptomyces avermitilis*.^1^, ^2^ *Streptomyces avermitilis* is a naturally occurring soil actinomycete.^3^ This EB stimulates high‐affinity GABA (Gamma Amino Butyric Acid) receptors and increases membrane chloride‐ion permeability.^1^, ^2^ Used as insecticides and pesticides, it is efficacious against many organisms like armyworm species, diamondback moth (*Plutellaxylostella*), cabbage looper (*Trichoplusiani* [Hubner]), beet armyworm (*Spodopteraexigua* (Hubner), etc..^2^ To the best our knowledge, there has been few available literatures regarding EB poisoning in human, and that too lacks specific recommendations to tackle cases of EB poisoning.

Here, we report a case of EB poisoning in child resulting in gastrointestinal distress and confusion, which was managed with vigorous lavage, fluids, and supportive treatments.

## 2 CASE PRESENTATION

A six‐year‐old girl was brought to the Emergency Department (ED) of Kalaiya District Hospital, Bara, Nepal, with complaints of nausea, vomiting, and abdominal pain at 3:30 pm. It was accompanied by her parents who had noted her of being alert and playful after returning from school on the same day. The mother while doing her household chores noticed her playing and consuming *“JALJEERA”* (cumin powder used to fortify beverages) sachets with a glass of water after a regular evening snack. Following to which she started developing repeated bouts of nausea, vomiting, and abdominal pain in next half an hour to an hour and was then brought to ED by her parents. The thirst for the perplexed and not so concluding history of presenting illness got quenched with the multiple probing and discussions with the family members, making them reveal of having several empty sachets at her home. One of the sachets was discovered to be of *“LURA*” instead of *“JALJEERA”*. The *“LURA*” comes as a brand name of a sachet of “Emamectin Benzoate 5% SG” (soluble granules). The packaging of *“LURA”* was similar to a packet of *“JALJEERA”* that easily led the child to consume the sachet of “Emamectin Benzoate 5% SG”. A provisional diagnosis of Emamectin Benzoate Poisoning was made.

Oriented to time, place, and person on examination, she was awake and alert but mildly confused and irritable. Her Glassgow Coma Scale (GCS) score was 13/15 (E3V4M6). Her pulse rate was 94 beats per minute and regular. Her respiratory rate, axillary body temperature, and saturation were 22 per min., 98.4֯F, and 98% respectively. Pupils were isochoric with normal light reflex. The chest was clear on auscultation. Auscultation of the precordium did not reveal a murmur or any other added sound. The abdomen was soft, flat, and without tenderness or rebound tenderness.

The laboratory investigations revealed hemoglobin 13.8 mg/dL (reference range: 12–16 mg/dL), white blood cells count 7700 cells/mm3 (reference range: 4000–11,000 cells/mm^3^), platelet 170,000 cells/mm3 (reference range: 150000–400,000 cells/mm^3^), Na^+^ 136 mEq/L (reference range: 135–145 mEq/L), K^+^ 4.2 mEq/L (reference range: 3.3–5.3 mEq/L), serum blood‐urea‐nitrogen 12.3 mg/dL (reference range: 6–20 mg/dL), creatinine 0.9 mg/dL (reference range: 0.6–1.2 mg/dL), and random blood sugar 82 mg/dL (reference range: 60–100 mg/dL). The urinalysis was normal without any casts or crystals. The chest X‐ray was normal without any infiltrates or adenopathy. The ECG (electrocardiogram) showed normal sinus rhythm. The arterial‐blood‐gas analysis showed pH 7.38 (reference range: 7.35–7.45), PaCO2 42 mm Hg (reference range: 35–45 mm Hg), HCO3 25 mEq/L (reference range: 22–26 mEq/L), and serum lactate 0.8 mmol/L (reference range: 0.7–2.5 mmol/L).

The initial treatment procedure of gastric lavage with 2 L normal saline, 50 g activated charcoal, and 500 ml coconut oil. Adding to it, she was given an intravenous (IV) injection of pantoprazole (@ 20 mg IV stat), ketorolac (@ 15 mg slow IV stat), ondansetron (@ 2 mg IV stat), and hydrocortisone (@ 50 mg slow IV stat) for symptoms management. 30 min following gastric lavage and emergency observation for 6 h, she was shifted to the general medical ward on the same day.

In the medical ward, the nasogastric tube was continued in situ for 24 h. With IV maintenance fluids, she was kept nil per oral (NPO) for next 24 h. The medications pantoprazole (@ 20 mg IV once a day), and ondansetron (@ 2 mg IV three times a day) were continued on the first day withholding the hydrocortisone. The ketorolac (@ 50 mg IV) was planned to give when needed. Vitals (saturation, pulse and respiratory rate and urine output) and systemic examinations (neurological state, chest auscultations, and abdominal palpation) were assessed four‐hourly in the ward and were noted to be normal.

The next day of inpatient admission, her symptoms like nausea, vomiting, and abdominal pain improved, and she was more alert and playful. The nasogastric tube was removed, and IV fluids and medications were stopped. Regarding the diet, the oral sips were allowed, followed by liquid, which was gradually advanced to a semisolid and solid diet. Also, the oral medications (pantoprazole @ 40 mg once a day; domperidone @10 mg twice a day; and oral fluids prepared with sachet of oral rehydration solution, ORS @ 1 sachet/1 L water) were continued.

On the third day of admission, she was discharged with proper counseling on the same oral medications for 3 more days, and ORS, and advised to follow‐up after 1 week in the clinic.

In follow‐up, after 1 week, she did not have any presenting complaints. Vitals and systemic examinations were within the normal limit. Neuropsychiatric evaluation was done which was normal.

## 3 DISCUSSION

In this case, the patient (weight 20 kilogram, BMI: 15.66 kg/m2) ingested a packet of “*LURA”* (1 packet = 5 g, Emamectin Benzoate 5% w/w), that is, 250 mg/kg of EB. Clinical manifestations were limited to a mild disturbance of consciousness and gastrointestinal distress.

The clinical presentations of EB poisoning involve central nervous system dysfunction and gastrointestinal upset. However, mammalian species are less sensitive to it due to lower GABA affinity and relative impermeability of the blood–brain barrier.^1^, ^2^ In animals, there is substantial evidence of behavioral effects like changes in motor activity (tremors, incoordination, ataxia, and lethargy) and neuronal changes in the form of degeneration and vacuolation of the neuronal cytoplasm. The cytoplasmic vacuoles are associated with cellular debris, macrophages, and pyknotic nuclei^4^, ^5^ . A fatal case report in humans has been reported after consumption of 500 g of Emamectin Benzoate 5% SG due to pulseless ventricular tachycardia.^6^

As EB has no specific antidote, the management of this poisoning is always supportive and symptomatic. As it possesses GABA mimetic activity, the drugs that enhance GABA activity like benzodiazepines, barbiturates, and valproic acid should be avoided.^1^, ^7^, ^8^

There is paucity of literature regarding the complete spectrum of manifestations of EB poisoning in human. Similarly, there are no consensus recommendations for the management of EB poisoning. The EB is available as 5% SG. In view of its water solubility nature, we used both coconut oil and activated charcoal for gastric lavage. We assumed that the coconut oil might coat the gastric mucosa to delay the absorption of EB and may additionally prevent damage to the raw mucosa because of anti‐ulcer nature of coconut oil in some studies.^9^, ^10^ Similarly, the activated charcoal also could delay absorption by adsorption of toxins which is evident from multiple literatures.^11^, ^12^

In this case, apart from the gastric lavage, symptomatic and supportive management was provided. This was a mild case of poisoning in which the patient improved quickly. It is important to label and store chemicals like this safely to minimize harm by accidental ingestion.

## 4 CONCLUSION

In the case of Emamectin Benzoate poisoning, vigorous gastric lavage with both activated charcoal and coconut oil can improve the patient's outcome. The rest of the treatment is symptomatic and supportive. Unfortunately, there is no specific antidote for this poisoning.

## AUTHOR CONTRIBUTIONS

GKY was involved in management of patient and in the conception of the report, literature review, initial manuscript preparation, editing and submission. DKR, KM, BP, and ARD were involved in the manuscript critique and review, and final manuscript preparation. All authors have read and approved the final manuscript.

## FUNDING INFORMATION

This study did not receive any specific grant from funding agencies in the public, commercial, or not‐for‐profit sectors.

## CONFLICT OF INTEREST

The authors declare no conflicts of interest.

## ETHICAL APPROVAL

Not applicable.

## CONSENT

Written informed consent was obtained from the parent for publication of this case report. A copy of the written consent is available for review by the Editor‐in‐Chief of this journal on request.

## Data Availability

Not applicable

## References

[1] Yen TH , Lin JL . Acute poisoning with emamectin benzoate. J Toxicol Clin Toxicol. 2004;42(5):657‐661. 15462160 10.1081/clt-200026968

[2] Jansson R , Brown R , Cartwright B , Cox D , Dunbar D , Dybas R , et al. Emamectin Benzoate: A Novel Avermectin Derivative for Control of Lepidopterous Pests; 1997. https://www.researchgate.net/publication/265977575_Emamectin_benzoate_A_novel_avermectin_derivative_for_control_of_lepidopterous_pests

[3] Olanrewaju OS , Babalola OO . Streptomyces: implications and interactions in plant growth promotion. Appl Microbiol Biotechnol. 2019;103(3):1179‐1188. 30594952 10.1007/s00253-018-09577-y PMC6394478

[4] Armstrong R , MacPhee D , Katz T , Endris R . A field efficacy evaluation of emamectin benzoate for the control of sea lice on Atlantic salmon. Can Vet J. 2000;41(8):607‐612. 10945125 PMC1476239

[5] Takai K , Suzuki T , Kawazu K . Development and preventative effect against pine wilt disease of a novel liquid formulation of emamectin benzoate. Pest Manag Sci. 2003;59(3):365‐370. 12639056 10.1002/ps.651

[6] Godhiwala P , Nirmal A , Bakre A , Wanjari A , Kumar S . Acute Emamectin benzoate poisoning‐a fatal case report. J Evol Med Dent Sci. 2020;23:9‐169.

[7] Polc P . Enhancement of GABAergic inhibition: a mechanism of action of benzodiazepines, phenobarbital, valproate and L‐cycloserine in the cat spinal cord. Electroencephalogr Clin Neurophysiol Suppl. 1982;36:188‐198. 6130936

[8] Macdonald RL , Kelly KM . Antiepileptic drug mechanisms of action. Epilepsia. 1995;36(Suppl. 2):S2‐S12. 10.1111/j.1528-1157.1995.tb05996.x 8784210

[9] Meng J , Chen T , Zhao Y , et al. Study of the mechanism of anti‐ulcer effects of virgin coconut oil on gastric ulcer‐induced rat model. Arch Med Sci AMS. 2019;15(5):1329‐1335. 31572481 10.5114/aoms.2018.76943 PMC6764319

[10] Bajwa SJS , Bajwa SK , Kaur J , Singh K , Panda A . Management of celphos poisoning with a novel intervention: a ray of hope in the darkest of clouds. Anesth Essays Res. 2010;4(1):20‐24. 25885082 10.4103/0259-1162.69301 PMC4173337

[11] Silberman J , Galuska MA , Taylor A . Activated charcoal. StatPearls [Internet]. StatPearls Publishing; 2022 [cited 2022 Jun 15]. http://www.ncbi.nlm.nih.gov/books/NBK482294/ 29493919

[12] Chyka PA , Seger D , Krenzelok EP , Vale JA . American academy of clinical toxicology, european association of poisons centres and clinical toxicologists. position paper: single‐dose activated charcoal. Clin Toxicol Phila pa. 2005;43(2):61‐87. 10.1081/clt-200051867 15822758

